# Association between fibrinogen-to-albumin ratio and carotid intraplaque neovascularization on AngioPLUS in patients with asymptomatic carotid stenosis

**DOI:** 10.1016/j.clinsp.2025.100710

**Published:** 2025-07-10

**Authors:** Jun He, Jimei Xu, Shugang Cao, Yuan Feng, Jian Wang, Yan Yan, Fang Ma, Mingwu Xia, Qingsong Wang

**Affiliations:** a Department of Neurology, PLA Clinical College of Anhui Medical University, The 901st Hospital of the Joint Logistics Support Force of the Chinese People's Liberation Army, Hefei 230001, PR China; b The Fifth Clinical Medical College of Anhui Medical University, Hefei 230001, PR China; c Department of Neurology, Hefei Hospital Affiliated to Anhui Medical University, The Second People's Hospital of Hefei, Hefei 230001, PR China; d Department of Ultrasound, Hefei Hospital Affiliated to Anhui Medical University, The Second People's Hospital of Hefei, Hefei 230001, PR China

**Keywords:** Carotid plaque, Intraplaque neovascularization, Fibrinogen-to-albumin ratio, Angio planewave ultrasensitive imaging

## Abstract

**Introduction:**

The importance of the Fibrinogen-to-Albumin Ratio (FAR) as a novel inflammatory and thrombotic biomarker in the development of carotid Intraplaque Neovascularization (IPN) is not well understood. This study aims to investigate the relationship between FAR and carotid IPN characteristics in patients with asymptomatic carotid stenosis.

**Methods:**

We enrolled patients with carotid plaques for the AngioPLUS examination, and clinical and laboratory indicators were collected. Carotid IPN was evaluated by semi-quantitative visual grading of IPN and Intraplaque Microvascular Flow (IMVF). Binary logistic regression models were performed to determine potential associations between various variables and the presence of high IPN and IMVF.

**Results:**

In the study, a total of 187 patients were included, with 73 in the high IPN group and 98 in the high IMVF group. The IPN score was positively correlated with the IMVF grade, as indicated by a correlation coefficient of 0.815 (*p* < 0.001). A FAR value of 7.578 was the optimal cutoff value to differentiate high and low IPN as well as high and low IMVF. After adjusting for various factors, multivariate logistic regression models demonstrated that FAR was a strong predictor of the presence of high IPN and IMVF. Furthermore, a high FAR level was also significantly correlated with the presence of high IPN (OR = 2.81, 95 % CI 1.49‒3.30, *p* = 0.001) and high IMVF (OR = 2.55, 95 % CI 1.39‒4.68, *p* = 0.002) when the variable FAR grouping was included in the above models.

**Conclusion:**

Elevated FAR is closely correlated with the presence of high IPN and IMVF on AngioPLUS and may facilitate plaque vulnerability.

## Highlights

• AngioPLUS is an innovative microvascular Doppler ultrasound technique for the evaluation of carotid IPN.
• The IPN score is positively correlated with the IMVF grade on AngioPLUS.
• FAR exhibits a strong association with both carotid IPN scores and IMVF grades.

## Introduction

Carotid plaque is a primary cause of ischemic strokes; in-depth studies have shown that plaque-induced luminal narrowing is not the only factor contributing to ischemic stroke events, as plaque composition and vulnerability have also been closely related to ischemic stroke.^1^ The predominant components of vulnerable plaques include large lipid nuclei, thin or ruptured fibrous caps, intraplaque hemorrhage, neovascularization, and inflammatory cells. Koole et al.^2^ suggested that Intraplaque Neovascularization (IPN) can induce intraplaque hemorrhage and plaque rupture, and thus accelerate plaque growth, increasing plaque instability and related complications.

Angio Planewave Ultrasensitive Imaging (AngioPLUS) is an innovative microvascular Doppler ultrasound technique, which has been successfully used to distinguish benign and malignant thyroid nodules, breast masses, and prostate tumors, by evaluating their microvasculature.^3^^,^^4^ In recent years, the AngioPLUS technique has been applied to evaluate carotid IPN or end-of-extremity microcirculation,^5^^,^^6^ thus achieving non-invasive and accurate detection of carotid IPN and the visualization of Intraplaque Microvascular Flow (IMVF) signals without the need for contrast injection as in Contrast-Enhanced Ultrasound (CEUS). Further, semi-quantitative grading of carotid IPN helps to assess the vulnerability of carotid plaque.^6^

Identifying associated risk factors or biomarkers that influence the formation and development of carotid IPN is particularly important for guiding further interventions. Fibrinogen, an inflammation-related protein, is a major factor involved in blood viscosity and fibrin formation that has been associated with carotid plaque formation.^7^^,^^8^ Albumin inhibits the expression of vascular cell adhesion molecule-1 and promotes the scavenging of oxygen free radicals, thereby reducing the inflammatory response and endothelial cell apoptosis and acting as a vasoprotective agent.^9^ Previous studies have shown that the Fibrinogen Albumin Ratio (FAR), a novel inflammatory and thrombotic biomarker, is associated with the severity and clinical outcomes of coronary artery disease.^10^^,^^11^ However, the role of FAR in carotid IPN has not yet been fully elucidated. This study aims to evaluate the role of readily available inflammatory biomarkers fibrinogen, albumin, and the derived FAR in the formation of carotid IPN in patients with asymptomatic carotid stenosis. By doing so, the study may offer valuable insights into potential treatment options and diagnostic markers for those with carotid IPN.

## Methods

### Study design and patients

This was a cross-sectional study focusing on carotid IPN. Consecutive asymptomatic patients who underwent the AngioPLUS screening for carotid plaques at our hospital were enrolled between October 2020 and December 2022. The local Ethics Committee approved the study. Written informed consent was provided by all participating patients or their guardians. Inclusion criteria were the following: patients aged > 18-years with clear 2D ultrasound images of the carotid arteries; a diagnosis consistent with carotid plaque formation (plaque thickness ≥ 1.5 mm); completion of AngioPLUS for carotid plaques. Patients were excluded if they had undergone Carotid Endarterectomy (CEA) or Carotid Stenting (CAS), had a history of malignancy or a new diagnosis of malignancy, or failed to complete AngioPLUS due to various factors (e.g., obesity, inability to cooperate with breathing, abnormal carotid artery course, severe dementia, psychiatric factors, etc.).

### Clinical data

We collected and recorded detailed demographic information (age, gender), cerebrovascular risk factors (such as current smoking, current drinking, hypertension, diabetes, dyslipidemia, etc.), Body Mass Index (BMI), and blood pressure (both systolic and diastolic) at admission for each patient. These variables were thoroughly documented to investigate their potential associations with carotid IPN.

### Laboratory indicators

We collected blood samples from all participants in the early morning after an overnight fast. These samples were subjected to routine blood tests, biochemical analyses, and coagulation parameter assessments. Blood tests were performed with the Sysmex XN-10 automated blood cell analyzer. Fasting Blood Glucose (FBG), Glycated hemoglobin (HbA1c), Triglycerides (TG), Total Cholesterol (TC), Low-Density Lipoprotein (LDL), albumin, uric acid, Homocysteine (HCY), and C-Reactive Protein (CRP) were measured using an automatic biochemical analyzer (HITACHI Automatic Analyzer 7600–020, Japan). Fibrinogen and d-dimer levels in plasma were measured with an automatic coagulation analyzer (Stago STAR Max, France). The Fibrinogen-to-Albumin Ratio (FAR) is calculated by the formula: FAR=Fibrinogen/albumin*10−2.

### Evaluation of carotid IPN

In this study, we performed a routine ultrasound examination along the carotid artery. When the carotid plaque was observed, we then conducted AngioPLUS to observe and evaluate the flow signals in the target plaque with the probe in terms of IPN and IMVF using the French Sonic SuperSonic Imagine AixPlorer Imaging Ultrasound Diagnostic Instrument (Sonic Red Series, built-in AP ultrasound imaging technology, equipped with SL10–2 probe). If a patient had bilateral carotid plaques, the higher-scoring side was selected as the final value.

Determining the IPN score and IMVF grade: In the AngioPLUS mode, IPN was identified by the short-line or strip-like hyperintense echo in a 2-minute video clip. The scoring standard of IPN is as follow:s^6^^,^^12^ 0 point, no blood flow signal in the plaque; 1 point, a small number of dotted or short line blood flow signals (<4) on one side of the plaque; 2 points, diffuse lines in the plaque dendritic or dendritic blood flow signals. The IMVF signals are categorized on a visual scale as follow:s^6^^,^^12^ Grade 0: no IMV signal within the plaque; Grade 1: moving IMVF limited to the adventitial side; Grade 2: IMVF moving to the plaque shoulder; Grade 3: IMVF moving to the core of the plaque; Grade 4: extensive IMVF. For clinical simplicity, we further categorized the IPN score as low IPN (score 0‒1) or high IPN (score 2) and the IMVF grade as low IMVF or (grade 0‒2) or high IMVF (grade 3‒4). The examinations mentioned above were evaluated by two trained sonologists using a double-blind method. If any disagreement arises, a third senior sonologist is consulted to resolve the issue. Moreover, the sonologists were unaware of the laboratory data for all patients.

### Statistical analysis

Statistical analysis was performed using SPSS 22.0 (SPSS Inc., Chicago, IL) software. Baseline clinical data and laboratory indicators were presented as mean ± standard deviation (mean ± SD) or median (inter-quartile range) for continuous variables, and percentage for categorical variables. Differences in continuous variables between groups were assessed by Student’s *t*-test or Mann-Whitney *U* test. Differences in categorical variables between groups were assessed by the χ^2^ test or Fisher’s exact test. Univariate and multivariate logistic regressions were performed to determine the associations between different variables and IPN score and IMVF grade. The Odds Ratio (OR) and 95 % Confidence Interval (95 % CI) were subsequently calculated. Spearman rank correlation analysis was used to determine the correlation between IPN scores and IMVF grades, and the correlation coefficient is expressed by *r*. A ROC curve was used to determine the optimal cut-off value for FAR to accurately identify high or low IPN and IMVF, and to calculate the corresponding sensitivity and specificity. Statistical significance was taken as *p* < 0.05. All plots were drawn using GraphPad Prism software (version 8.0).

## Results

By December 31, 2022, a total of 187 patients were eventually enrolled, including 109 males and 78 females, with a median age of 68 years for the study population. In terms of risk factors for vascular disease, there were 148 patients (79.1 %) with hypertension, 48 patients (25.7 %) with diabetes, and 30 patients (16.0 %) with dyslipidemia (Table 1). Ninety-nine patients had bilateral carotid plaques and 88 patients had unilateral carotid plaques. Among these patients, 114 were categorized as the low IPN group (score 0‒1) and 73 as the high IPN group (score 2) according to IPN score; while 89 were in the low IMVF group (grade 0‒2) and 98 in the high IMVF group (grade 3‒4) according to IMVF grade. The IPN score was positively correlated with IMVF grade with a correlation coefficient of *r* = 0.815 (*p* < 0.001).

**Table 1:** Clinical and laboratory indicators compared between high and low FAR groups.

Variables	All patients (*n* = 187)	Low FAR (*n* = 97)	High FAR (*n* = 90)	p-value
Age (years)	68 (12)	68 (14)	71 (11)	0.004
Male, n ( %)	109 (58.3)	61 (62.9)	48 (53.3)	0.186
Hypertension, n ( %)	148 (79.1)	79 (81.4)	69 (76.7)	0.422
Diabetes mellitus, n ( %)	48 (25.7)	24 (24.7)	24 (26.7)	0.763
Dyslipidemia, n ( %)	30 (16.0)	19 (19.6)	11 (12.2)	0.170
Current drinking, n ( %)	36 (19.3)	21 (21.6)	15 (16.7)	0.388
Current smoking, n ( %)	43 (23.0)	26 (26.8)	17 (18.9)	0.199
BMI (Kg/m^2^)	24.09±2.81	24.01±2.67	24.17±2.95	0.685
SBP (mmHg)	141 (21)	142 (16)	141 (24)	0.735
DBP (mmHg)	89 (13)	90 (14)	86 (13)	0.146
WBC (*10^9/L),	6.26 (2.39)	6.24 (2.12)	6.29 (2.73)	0.655
Neutrophils ( %)	60.1 (13.1)	59.2 (13.3)	61.5 (12.8)	0.162
Lymphocyte ( %)	29.2 (12.0)	31.4 (11.0)	28.0 (12.1)	0.028
Platelet (*10^9/L)	167 (70)	161 (63)	175 (79)	0.218
FBG (mmoL/L)	5.2 (1.6)	5.1 (1.3)	5.4 (1.7)	0.043
HbA1c ( %)	5.7 (1.4)	5.8 (1.2)	6.1 (1.3)	0.032
TC (mmoL/L)	4.30±1.02	4.28±1.07	4.32±0.96	0.781
TG (mmoL/L)	1.41 (1.09)	1.42 (1.12)	1.37 (1.01)	0.521
LDL (mmoL/L)	2.45±0.77	2.43±0.82	2.48±0.74	0.648
CRP (mg/L)	3.6 (6.8)	3.0 (4.2)	4.5 (7.1)	0.012
HCY (μmoL/L)	13.5 (6.1)	12.7 (5.5)	14.2 (6.5)	0.026
Uric acid (μmoL/L)	335 (106)	320 (114)	342 (106)	0.161
D-dimmer (μg/mL)	0.35 (0.33)	0.32 (0.23)	0.45 (0.55)	<0.001

Note: Numbers are given as mean ± Standard Deviation (SD), median (inter-quartile range) for continuous variables, and percentage for categorical variables.

FAR, Fibrinogen-to-Albumin Ratio; BMI, Body Mass Index; SBP, Systolic Blood Pressure; DBP, Diastolic Blood Pressure; WBC, White Blood Cell; FBG, Fasting Blood Glucose; HbA1c, Glycated Haemoglobin; TG, Triglycerides; TC, Total Cholesterol; LDL, Low-Density Lipoprotein; HCY, Homocysteine; CRP, and C-Reactive Protein.

### Relationship between FAR and IPN score as well as IMVF grade

**Fig. 1:**
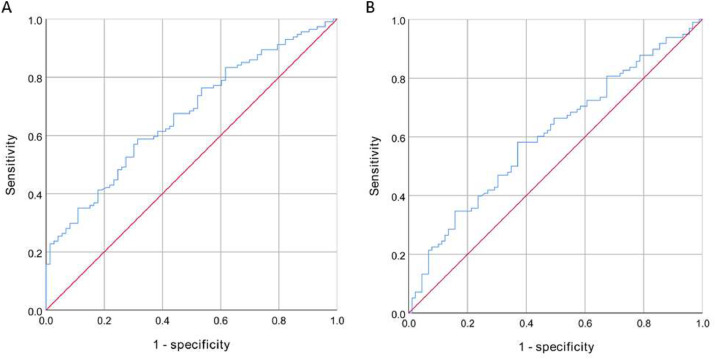
The Receiver Operating Curve (ROC) shows the predictive value of FAR for carotid IPN (A) and IMVF (B).

The high IPN group had higher levels of fibrinogen (*p* < 0.001) and FAR (*p* < 0.001) compared to the low IPN group, while the difference in albumin levels between the two groups was not statistically significant (*p* = 0.155). A FAR value of 7.578 was the optimal cutoff value to distinguish between high and low IPN, and the area under the ROC curve was 0.67 (95 % CI 0.59‒0.75, *p* < 0.001). The FAR exhibited a sensitivity of 58.8 % and a specificity of 68.5 % for discriminating between high and low IPN (Fig. 1A).

The FAR levels were significantly higher in the high IMVF group compared to the low IMVF group (*p* = 0.041). However, the differences in albumin and fibrinogen levels between the two groups were not statistically significant. The optimal cutoff value to differentiate high and low IMVF was also 7.578, with the area under the ROC curve of 0.61 (95 % CI 0.53‒0.69, *p* = 0.013). The FAR exhibited a sensitivity of 58.2 % and a specificity of 62.9 % for discriminating between high and low IMVF (Fig. 1B).

### Comparison of clinical and laboratory indicators between high and low FAR groups

**Fig. 2:**
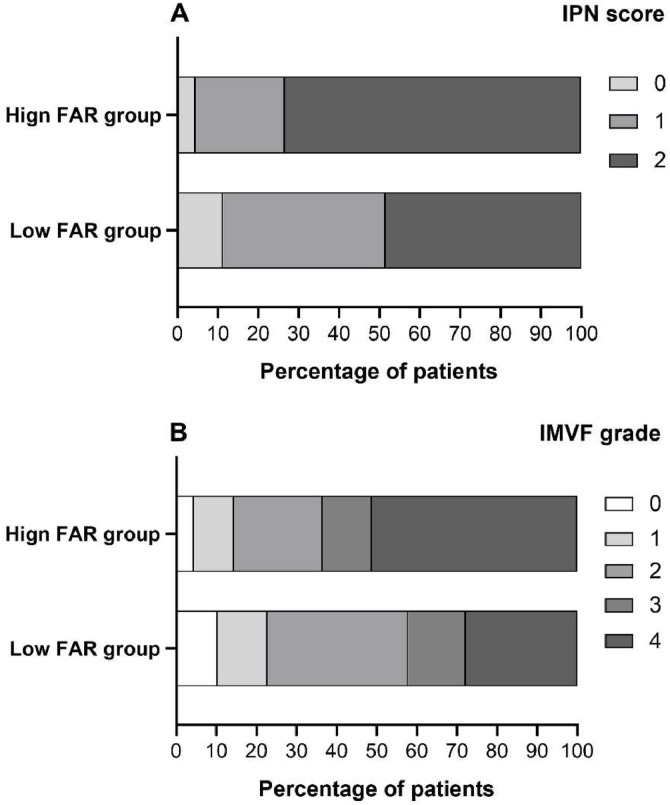
The IPN score (A) and IMVF grade (B) distribution between the high and low FAR groups.

Using a FAR cutoff value of 7.578, we categorize patients into two groups: a low FAR group (FAR < 7.578) and a high FAR group (FA *R* ≥ 7.578). The characteristics of these two groups are presented in Table 1 and Fig. 2. Patients in the high FAR group were older (*p* = 0.010) and had higher levels of FBG (*p* = 0.043), HbA1c (*p* = 0.032), CRP (*p* = 0.012), HCY (*p* = 0.026), and d-dimer (*p* < 0.001), while showing a lower lymphocyte ratio (*p* = 0.028) than the low FAR group (Table 1).

### The FAR was associated with the presence of high IPN and IMVF

We used binary logistic regression models to analyze the association between different variables and the presence of high IPN. Our results demonstrated that age, fibrinogen, HbA1c, and FAR were strongly correlated with the presence of high IPN (with all p-values <0.05) (Tables 2 and 3). In addition, high FAR was also significantly correlated with the presence of high IPN (OR = 2.81, 95 % CI 1.49‒3.30, *p* = 0.001) when the variable FAR grouping was included in the above models.

**Table 2:** Univariate logistic regression analysis of associated factors for high IPN.

Variables	Low IPN group (*n* = 114)	High IPN group (*n* = 73)	Unadjusted OR (95 % CI)	p-value
Age (years)	67 (13)	70 (12)	1.03 (1.00‒1.06)	0.040
Male, n ( %)	40 (54.8)	69 (60.5)	1.27 (0.70‒2.29)	0.438
Hypertension, n ( %)	54 (74.0)	94 (82.5)	1.65 (0.81‒3.37)	0.166
Diabetes mellitus, n ( %)	14 (19.2)	34 (29.8)	1.79 (0.88‒3.63)	0.106
Dyslipidemia, n ( %)	9 (12.3)	21 (18.4)	1.61 (0.69‒3.73)	0.271
Current drinking, n ( %)	10 (13.7)	26 (22.8)	1.86 (0.84‒4.13)	0.127
Current smoking, n ( %)	13 (17.8)	30 (26.3)	1.65 (0.79‒3.42)	0.180
BMI (Kg/m^2^)	23.78±2.78	24.28±2.82	1.07 (0.96‒1.19)	0.236
SBP (mmHg)	140 (16)	142 (24)	1.00 (0.99‒1.02)	0.921
DBP (mmHg)	89 (13)	89 (14)	0.99 (0.97‒1.01)	0.444
WBC (*10^9/L),	6.24 (2.18)	6.35 (2.57)	1.06 (0.93‒1.22)	0.381
Neutrophils ( %)	59.8 (14.1)	60.8 (12.6)	1.01 (0.98‒1.04)	0.614
Lymphocyte ( %)	31.4 (10.2)	28.4 (12.0)	0.97 (0.94‒1.01)	0.087
Platelet (*10^9/L)	165 (61)	169 (87)	1.00 (0.99‒1.01)	0.994
FBG (mmoL/L)	5.1 (1.3)	5.3 (1.8)	1.08 (0.94‒1.23)	0.274
HbA1c ( %)	5.5 (1.0)	6.1 (1.3)	1.53 (1.15‒2.04)	0.004
TC (mmoL/L)	4.32±1.10	4.28±0.96	0.96 (0.72‒1.29)	0.795
TG (mmoL/L)	1.33 (1.12)	1.46 (1.07)	0.95 (0.78‒1.15)	0.581
LDL (mmoL/L)	2.46±0.83	2.44±0.74	0.98 (0.67‒1.43)	0.909
CRP (mg/L)	3.6 (5.2)	3.4 (7.6)	1.04 (0.98‒1.09)	0.179
HCY (μmoL/L)	12.7 (5.2)	14.2 (6.1)	0.99 (0.96‒1.02)	0.426
Uric acid (μmoL/L)	326 (129)	342 (100)	1.00 (1.00‒1.01)	0.478
Fibrinogen (g/L)	2.91 (0.66)	3.18 (0.91)	3.15 (1.76‒5.62)	<0.001
D-dimmer (μg/mL)	0.34 (0.24)	0.39 (0.37)	3.86 (1.26‒11.79)	0.018
Albumin (g/L)	42.1 ± 3.5	41.4 ± 3.4	0.94 (0.86‒1.02)	0.156
FAR (10^–2^)	6.99 (1.87)	7.84 (2.229)	1.54 (1.24‒1.92)	<0.001

Note: Numbers are given as mean ± Standard Deviation (SD), median (inter-quartile range) for continuous variables, and percentage for categorical variables.

IPN, Intraplaque Neovascularization; OR, Odds Ratio; 95 % CI, 95 % Confidence Interval; BMI, Body Mass Index; SBP, Systolic Blood Pressure; DBP, Diastolic Blood Pressure; WBC, White Blood Cell; FBG, Fasting Blood Glucose; HbA1c, Glycated Haemoglobin; TG, Triglycerides; TC, Total Cholesterol; LDL, Low-Density Lipoprotein; HCY, Homocysteine; CRP, and C-Reactive Protein; FAR, Fibrinogen-to-Albumin Ratio.

**Table 3:** Multivariate logistic regression models of associated factors for high IPN.

Models	Adjusted OR (95 % CI)	p-value
Model 1 (with Fibrinogen)		
Fibrinogen	2.90 (1.61‒5.24)	<0.001
HbA1c	1.46 (1.08‒1.96)	0.013
Model 2 (with Albumin)		
Age	1.04 (1.01‒1.07)	0.022
HbA1c	1.55 (1.17‒2.05)	0.002
Model 3 (with FAR)		
FAR	1.51 (1.21‒1.88)	<0.001
HbA1c	1.47 (1.09‒1.98)	0.011

Note: Potential risk factors with a p-value of < 0.1 in the univariate analysis or clinically relevant factors were included in the multivariate logistic regression models with a forward stepwise procedure.

Model 1: Age + dyslipidemia + Lymphocyte + HbA1c + *d*-dimmer + Fibrinogen.

Model 2: Age + dyslipidemia + Lymphocyte + HbA1c + *d*-dimmer + Albumin.

Model 3: Age + dyslipidemia + Lymphocyte + HbA1c + *d*-dimmer +FAR.

OR, Odds Ratio; 95 % CI, 95 % Confidence Interval; HbA1c, Glycated Haemoglobin; FAR, Fibrinogen-to-Albumin Ratio.

In the same way, we analyzed the association between different variables and the presence of high IMVF. Our results showed that hypertension, fibrinogen, and FAR were significantly associated with the presence of high IMVF (with all p-values <0.05) (Tables 4 and 5). In addition, high FAR was also significantly correlated with the presence of high IMVF (OR = 2.55, 95 % CI 1.39‒4.68, *p* = 0.002) when the variable FAR grouping was included in the above models.

**Table 4:** Univariate logistic regression analysis of associated factors for high IMVF.

Variables	Low IMVF group (*n* = 89)	High IMVF group (*n* = 98)	Unadjusted OR (95 % CI)	p-value
Age (years)	68 (11)	71 (12)	1.03 (1.00‒1.06)	0.093
Male, n ( %)	46 (51.7)	63 (64.3)	1.68 (0.94‒3.02)	0.082
Hypertension, n ( %)	64 (71.9)	84 (85.7)	2.34 (1.13‒4.87)	0.022
Diabetes mellitus, n ( %)	21 (23.6)	27 (27.6)	1.23 (0.64‒2.38)	0.537
Dyslipidemia, n ( %)	13 (14.6)	17 (17.3)	1.23 (0.56‒2.70)	0.610
Current drinking, n ( %)	13 (14.6)	23 (23.5)	1.79 (0.85‒3.80)	0.128
Current smoking, n ( %)	18 (20.2)	25 (25.5)	1.35 (0.68‒2.69)	0.392
BMI (Kg/m^2^)	24.12±2.82	24.06±2.80	0.99 (0.90‒1.10)	0.878
SBP (mmHg)	140 (19)	144 (24)	1.01 (1.00‒1.03)	0.137
DBP (mmHg)	86 (13)	90 (14)	1.01 (0.98‒1.03)	0.689
WBC (*10^9/L),	6.24 (2.38)	6.35 (2.53)	0.97 (0.85‒1.10)	0.594
Neutrophils ( %)	59.9 (13.8)	60.6 (11.9)	0.99 (0.96‒1.02)	0.453
Lymphocyte ( %)	31.0 (12.0)	28.4 (11.7)	0.99 (0.96‒1.03)	0.621
Platelet (*10^9/L)	167 (63)	169 (81)	1.00 (1.00‒1.01)	0.693
FBG (mmoL/L)	5.2 (1.7)	5.1 (1.5)	0.94 (0.83‒1.06)	0.315
HbA1c ( %)	5.7 (1.3)	6.1 (1.1)	1.07 (0.88‒1.30)	0.508
TC (mmoL/L)	4.45±1.04	4.16±0.98	0.76 (0.57‒1.01)	0.060
TG (mmoL/L)	1.41 (1.24)	1.45 (0.98)	0.91 (0.73‒1.13)	0.387
LDL (mmoL/L)	2.53±0.80	2.38±0.75	0.52 (0.18‒1.53)	0.233
CRP (mg/L)	3.8 (7)	3.3 (5.8)	1.00 (0.98‒1.02)	0.707
HCY (μmoL/L)	12.7 (5)	14.3 (6.4)	0.99 (0.96‒1.02)	0.608
Uric acid (μmoL/L)	326 (111)	342 (104)	1.00 (1.00‒1.01)	0.396
Fibrinogen (g/L)	3.03 (0.67)	3.17 (0.85)	1.50 (0.97‒2.32)	0.068
D-dimmer (μg/mL)	0.34 (0.27)	0.36 (0.32)	1.07 (0.72‒1.58)	0.734
Albumin (g/L)	41.94±3.43	41.37±3.44	0.95 (0.88‒1.04)	0.265
FAR (10^–2^)	7.09 (1.81)	7.79 (2.07)	1.18 (1.01‒1.39)	0.041

Note: Numbers are given as mean ± Standard Deviation (SD), median (inter-quartile range) for continuous variables, and percentage for categorical variables.

IMVF, Intraplaque Microvascular Flow; OR, Odds Ratio; 95 % CI, 95 % Confidence interval; BMI, Body Mass Index; SBP, Systolic Blood Pressure; DBP, Diastolic Blood Pressure; WBC, White Blood Cell; FBG, Fasting Blood Glucose; HbA1c, Glycated Haemoglobin; TG, Triglycerides; TC, Total Cholesterol; LDL, Low-Density Lipoprotein; HCY, Homocysteine; CRP, and C-Reactive Protein; FAR, Fibrinogen-to-Albumin Ratio.

**Table 5:** Multivariate logistic regression models of associated factors for high IMVF.

Models	Adjusted OR (95 % CI)	p-value
Model 1 (with Fibrinogen)		
Fibrinogen	1.59 (1.02‒2.46)	0.039
Hypertension	2.60 (1.22‒5.51)	0.013
Model 2 (with Albumin)		
Hypertension	2.34 (1.13‒4.87)	0.022
Model 3 (with FAR)		
FAR	1.22 (1.03‒1.43)	0.020
Hypertension	2.71(1.27‒5.78)	0.010

Note: Potential risk factors with a p-value of <0.1 in the univariate analysis or clinically relevant factors were included in the multivariate logistic regression models with a forward stepwise procedure.

Model 1: Age + Male + Hypertension + dyslipidemia + TC + Fibrinogen.

Model 2: Age + Male + Hypertension + dyslipidemia + TC + Albumin.

Model 3: Age + Male + Hypertension + dyslipidemia + TC +FAR.

OR, Odds Ratio; 95 % CI, 95 % Confidence Interval; FAR, Fibrinogen-to-Albumin Ratio.

## Discussion

The present study found that FAR exhibited a strong association with both carotid IPN scores and IMVF grades on AngioPLUS. Additionally, HbA1c was found to be associated with carotid IPN score, while hypertension was correlated with IMVF grade. Therefore, elevated FAR could potentially indicate a higher severity and wider distribution of carotid IPN thus serving as an indicator worth monitoring.

IPNs are new capillaries generated from pre-existing vessels in budding and non-budding forms by endothelial cell proliferation and migration on top of the pre-existing capillaries.^13^ Vasa vasorum provides nourishment to the vessel walls. Most IPNs mainly originate from the adventitia and less often from the main vessel lumen. Extending vasa vasorum to the full thickness of the media and intima of atherosclerotic segments represents pathological neovascularization.^14^ Growing experimental evidence has identified neovascularization from the adventitial vasa vasorum and induced intraplaque hemorrhage as key indicators during the development of vulnerable atherosclerotic plaques.^15^^,^^16^ IPN located at the fibrous cap and shoulders on CEUS is a reliable indicator for identifying plaque vulnerability,^17^ and carotid IPN on CEUS is an independent predictor of future ischemic stroke events in patients with recent ischemic stroke.^17^^,^^18^ In the study by Cui et al.,^18^ carotid IPN was graded from 0 to 2 according to the extent of the microbubbles on CEUS. After 30±6 months of follow-up in 76 patients, 30 experienced subsequent vascular events, and a carotid IPN score of 2 on CEUS remained an independent predictor of subsequent vascular events (OR = 6.066, 95 % CI 1.565‒23.512) in multifactorial logistic regression analysis. Therefore, accurate evaluation of carotid IPN characteristics and identification of associated risk factors are of essential importance to guide its intervention and reduce the occurrence of future ischemic events.^19^

Fibrinogen is one of the key factors of the coagulation response that participates in forming atheromatous plaques. It is already elevated in the early stages of atherosclerosis and deposited in the vessel wall before LDL.^20^ Fibrinogen and its degradation products participate in the early stages of atherosclerosis due to the stimulation of smooth muscle cell proliferation and migration. The migration and proliferation of vascular smooth muscle from the middle to the inner layer and the adhesion of fibrinogen and fibrin-binding substances are essential in atherosclerosis and thrombosis. In addition, fibrin promotes lipoprotein adsorption to the intima and increases lipid accumulation in fibrous plaques.^21^^,^^22^ These processes constantly exacerbate the process of atherosclerosis, thus contributing to plaque formation, progression, and rupture, and increasing their vulnerability, where the inflammatory response triggered by fibrinogen plays a key role. Previous studies have shown that an increase in fibrinogen is associated with both the degree of coronary atherosclerosis and carotid plaque formation.^7^^,^^10^^,^^11^ The present study suggests that a high fibrinogen level is also an independent risk factor for the formation and distribution of carotid IPN. Albumin is a key protein in human serum, which has platelet aggregation inhibitory and antioxidant properties and is inversely related to inflammation.^23^ Decreased albumin can increase endothelial cell vascular cell adhesion molecule 1 activity and reduce anti-inflammatory effects, thereby increasing the concentration of free lysophosphatidylcholine, stimulating lipid and coagulation factor synthesis, and promoting the formation of atherosclerotic plaque and thrombosis.^23^^,^^24^ Our results also showed that the albumin level was decreased in the high IPN group compared to the low IPN group (OR = 0.94), although the difference was not statistically significant (*p* = 0.156). However, the derived FAR from them was significantly associated with both carotid IPN score and IMVF grade, suggesting that FAR may provide more comprehensive information and better predict carotid IPN characteristics. Previous studies have shown that FAR is associated with the severity and prognosis of several diseases including coronary artery disease,^10^^,^^11^ pontine infarction,^25^ sleep apnea,^26^ etc. Therefore, these inflammatory biomarkers are not just bystanders to ongoing inflammation but have a potential role in the pathophysiology of carotid atherosclerosis. The use of readily available inflammatory biomarkers fibrinogen, albumin, and the derived FAR allows us to more effectively identify these patients at high risk of carotid plaque and monitor the efficacy of pharmacologic treatments.^22^

In addition, we found that HbA1c and hypertension were associated with carotid IPN. Previous studies have also confirmed the relationship between diabetes and carotid plaque formation.^27^^,^^28^ Magnoni et al. suggested that intraplaque neovascularization frequently occurred in asymptomatic patients with intermediate carotid stenosis and was more prevalent in those with diabetes.^29^ Although we did not identify an association between diabetes and carotid IPN, HbA1c was found to be significantly associated with it, thus further suggesting that poor glycemic control is a key factor in the formation of carotid IPN. In their study, Daida et al. investigated the association between HbA1c and plaque regression, suggesting that plaque regression was less pronounced in patients with high HbA1c levels compared with those with low levels.^30^ The association between hypertension and carotid plaque formation is well established^31^^,^^32^; however, there are fewer studies on the relationship between hypertension and carotid IPN. The study by Tan et al. indicated that H-type hypertension (OR = 3.036, 95 % CI 1.258‒7.329) was independently connected with the degree of plaque enhancement on CEUS even after adjusting for other covariates and might facilitate plaque vulnerability.^33^ In the present study, we found hypertension was associated with the IMVF grade of the carotid IPN.

Currently, there are limited studies that report on the association between FAR and carotid IPN. This study analyzed in detail the correlation between FAR and carotid IPN characteristics and confirmed that FAR was associated with the severity and distribution of carotid IPN on AngioPLUS. However, the present study has several limitations. First, this was a single-center observational study with limited sample size and geographical limitations, which may have some selection bias. Accordingly, further, preferably multi-center studies with a larger sample are needed to validate reported findings. Second, we did not use CEUS, the gold standard for diagnosing carotid IPN, to evaluate its characteristics, mainly due to the invasive nature of CEUS. Third, nutritional status was not assessed at admission, and nutritional status assessment may help to understand the role of albumin and FAR in the evaluation of carotid IPN. Fourth, we did not dynamically evaluate whether fiber-lowering treatment for patients with high fibrinogen levels would reduce the severity of carotid IPN, which future studies should also address. Finally, the mechanisms of how FAR is involved in carotid IPN formation have not been fully elucidated. Therefore, future basic research is needed to fully elucidate this issue.

## Conclusion

Elevated FAR is closely correlated with the presence of high IPN and IMVF on AngioPLUS.

## Ethics approval and consent to participate

This study was approved by the Institutional Review Board of the hefei Hospital Affiliated to Anhui medical University (2022077) . The current study was carried out according to the Declaration of Helsinki and following the STARD guidelines. Written informed consent was provided by all participating patients or their guardians.

## Funding

This study was supported by grants from the Applied Medicine Research Project of Hefei Municipal Health Commission (Hwk2022zd004).

## Declaration of competing interest

The authors declare no conflicts of interest.

## Data Availability

All data generated for this study are included in the article. The datasets generated during the current study are available from the corresponding author upon reasonable request.
